# A Supportive Group Intervention for Caregivers to Patients Diagnosed With Glioblastoma: Protocol for the SUGRI Study

**DOI:** 10.2196/81948

**Published:** 2026-03-11

**Authors:** Sara Nordentoft, Rikke Guldager, Helle Pappot, Tiit Mathiesen, Anders Tolver, Karin Piil

**Affiliations:** 1 Copenhagen University Hospital, Copenhagen, Denmark and Department of Clinical Medicine, University of Copenhagen, Copenhagen, Denmark. Copenhagen Ø Denmark; 2 Copenhagen University Hospital, Copenhagen, Denmark. Dept. of People and Technology, Roskilde University, Roskilde, Denmark Copenhagen University Hospital Denmark; 3 Copenhagen University Hospital and University of Copenhagen, Copenhagen, Denmark and Department of clinical medicine, University of Copenhagen, Copenhagen, Denmark copenhagen Denmark; 4 Department of Neurosurgery, Copenhagen University Hospital; Department of clinical medicine, University of Copenhagen, Copenhagen, Denmark; Department of Clinical Neuroscience, Karolinska Institutet, Stockholm, Sweden Copenhagen Ø Denmark; 5 Statistics and Data Analysis, Danish Cancer Instititute, Danish Cancer Institute, Copenhagen, Denmark Copenhagen Ø Denmark; 6 Copenhagen University Hospital, Copenhagen and Department of clinical medicine, University of Copenhagen, Denmark Copenhagen Denmark; 7 Curtin University Perth, Western Australia Australia

**Keywords:** caregiver, glioblastoma, group intervention, high-grade glioma, neuro-oncology, supportive care

## Abstract

**Background:**

Caregivers to a person diagnosed with a glioblastoma often face significant responsibilities, balancing the demands of care with the complexities of the disease and treatment trajectory, while also coping with concerns and uncertainty of the future. Caregivers report unmet needs of information and support throughout the patient’s disease and treatment trajectory, and they may benefit from targeted supportive care interventions.

**Objective:**

The aim of this study is to develop, test, and evaluate the feasibility of a supportive group intervention among caregivers to patients diagnosed with a glioblastoma.

**Methods:**

The study consists of 3 phases with ongoing patient and public involvement (PPI). In the first phase, a systematic review will be carried out exploring the outcome of supportive group interventions for caregivers to patients diagnosed with a primary brain tumor. In the second phase, the design and development of the intervention will be conducted in cooperation with a panel of caregivers using PPI process. The third phase will include the feasibility and evaluation of the intervention. The study will be guided by the British Medical Research Council’s framework for developing complex interventions. The feasibility of the intervention will be tested in reference to relevant parameters. Quantitative data in terms of questionnaires will be analyzed using descriptive statistics, and qualitative evaluation interviews will be analyzed using thematic analysis.

**Results:**

A national panel of caregivers (N=10) has been established, and the final design of the intervention is currently developed through an ongoing PPI process. A total of 3 hospitals in Denmark have committed to participating in the recruitment for the feasibility study. Recruitment began in April 2025, and the SUGRI (Supportive Group Intervention for caregivers to patients diagnosed with a glioblastoma) intervention will undergo a feasibility assessment in a multicenter study starting in August 2025, with the final evaluation planned for April 2026.

**Conclusions:**

This study is expected to provide necessary insights to guide caregiver initiatives, ultimately improving support for caregivers within the neuro-oncology field. A supportive group intervention offered to the caregivers has the potential to address specific caregiver needs and strengthen their supportive network. Providing caregivers with support may enhance their perceived support, strengthen family functioning, and provide them with strategies to manage caregiving challenges, ultimately benefiting both caregivers and patients.

**Trial Registration:**

ClinicalTrials.gov NCT06869577; https://clinicaltrials.gov/study/NCT06869577

**International Registered Report Identifier (IRRID):**

PRR1-10.2196/81948

## Introduction

Primary malignant brain tumors are among the most fatal cancers and have an international incidence rate of 6.89 per 100,000, compared with 7.03 per 100,000 in Denmark [1,2]. The most common malignant brain tumor is a glioblastoma (GBM), accounting for 51.5% of all malignant brain tumors. It is associated with a poor prognosis: a 5-year relative survival rate of 5% [1]. The typical disease trajectory of 12 to 15 months includes initial surgical intervention (resection or biopsy), followed by a 9- to 10-month standard treatment plan consisting of concomitant chemotherapy or radiation therapy, with subsequent adjuvant chemotherapy of temozolomide [3]. Despite aggressive treatment, tumor progression occurs almost inevitably [4].

The diagnosis of a GBM is usually preceded by symptoms such as an epileptic seizure, focal neurological deficits, or cognitive changes that may affect the patient’s functional status [2]. Patients often develop a complex symptom burden, including physical symptoms such as aphasia and hemiparesis and psychological symptoms such as cognitive deficits, anxiety, distress, and depression. The symptoms may change as tumor progresses [5-7]. The combination of a rapid onset of the disease, the intense disease and treatment trajectories, and the uncertainty of the future affect not only the individual but also those close to them who function as informal caregivers, a role that often demands great caring responsibilities [8,9]. The caregiver burden is described as multidimensional with components affecting caregivers physically, emotionally, socially, and economically [10]. Caregivers express being unprepared for the new role and report symptoms of anxiety and depression [4,9]. Additionally, social isolation is often a consequence of the caregiver role. Furthermore, the *invisibility* of the patients’ symptoms can lead to withdrawal of support from family and previous networks [4,11]. Caregivers emphasize the importance of meeting other caregivers in similar situations so that they can share experiences and recognize emotions or concerns in a peer-to-peer environment [12,13]. Peer support may represent an important access to information, advice, and practical help for caregivers and address caregivers’ individual needs of support [12,14,15]. It is often a struggle for caregivers to be separated from the patient, as they worry about risk of seizures or cognitive symptoms that necessitate constant supervision, a struggle that can be alleviated with support [16]. Online access to peer support and information may be valuable for caregivers. Such a tool is accessible and has the potential to ensure broad reach while remaining scalable at the national level. Moreover, online support aligns well with the recommendations of the International Council of Nurses, which indicate that digital health can address population needs, strengthen health systems, and remain feasible despite shortages in the health workforce [17].

Several authors address the lack of longitudinal studies and advocate for ongoing supportive interventions to target caregivers of patients diagnosed with a brain tumor [10,18,19]. A need of such interventions is further echoed in international research agendas [20,21]. A systematic review from 2021 concluded feasibility of interventions for people with a brain tumor and their caregivers [14]. Importantly, support groups exclusively for caregivers may fulfill their need for peer support [14,22]. Therefore, this study aims to develop, test, and evaluate the feasibility of a supportive group intervention among caregivers to patients diagnosed with a GBM.

## Methods

### Study Design

**Figure 1:**
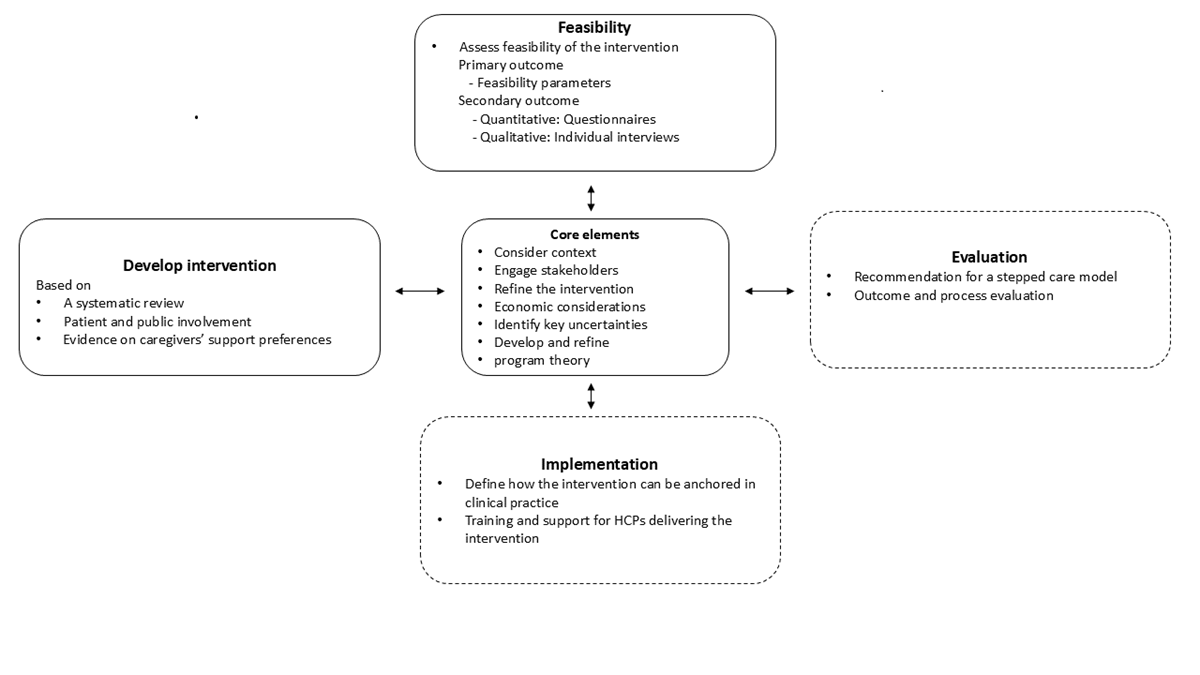
Complex intervention for the SUGRI (Supportive Group Intervention for caregivers to patients diagnosed with a glioblastoma) study. Solid lines represent this study’s activities; dashed lines represent planned future activities. HCP: health care professional.

**Figure 2:**
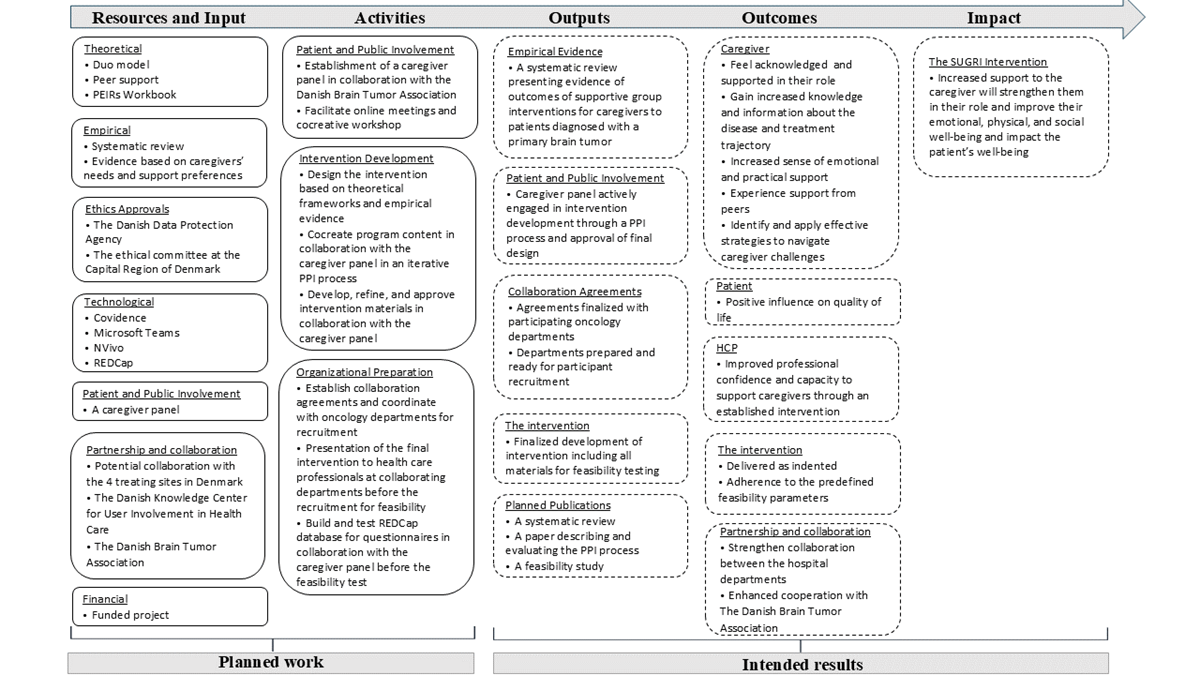
Logic model. Solid lines represent planned work; dashed lines represent intended results. HCP: health care professional; PEIR: Patient Engagement in Research; PPI: patient and public involvement; REDCap: Research Electronic Data Capture; SUGRI: Supportive Group Intervention for caregivers to patients diagnosed with a glioblastoma.

This study titled SUGRI (Supportive Group Intervention for caregivers to patients diagnosed with a glioblastoma) is a 3-phase study. The overall framework is guided by the British Medical Research Council (MRC) framework for developing complex intervention. This methodological framework entails 4 main phases of intervention research: development or identification of the intervention, feasibility, evaluation, and implementation. In this study, we develop, test, and evaluate the feasibility of an intervention; therefore, we are primarily guided by the first 2 phases of the MRC framework: the development phase and the feasibility phase [23-25]. The phases of the MRC framework applied to the SUGRI study are presented in Figure 1. An overview of the intervention logic, including planned activities and expected outcomes, is presented in Figure 2. A patient and public involvement (PPI) plan will be carried out throughout all phases of the SUGRI study, guided by the workbook to guide the development of a Patient Engagement in Research plan [26]. The recommended checklist for research protocols, SPIRIT (Standard Protocol Items: Recommendations for Interventional Trials), has been applied (Multimedia Appendix 1) [27].

### Study Population and Eligibility Criteria

The term “caregivers” is in this study defined according to the criteria provided by The National Cancer Institute [28]: “Informal caregiving is broadly defined as services provided by an unpaid person, such as helping with personal needs and household chores, managing a person’s finances, arranging for outside services, or visiting regularly. An informal caregiver is usually a relative or friend who may or may not live in the same household as the person with cancer who requires care.” Inclusion criteria are family caregivers (aged ≥18 years) to patients (aged ≥18 years) diagnosed with a GBM grade 4 or diffuse astrocytoma grade 4 receiving chemoradiotherapy treatment. Despite differential terminology of GBM grade 4 and diffuse astrocytoma grade 4 since 2021 [29], the study will include both categories of grade 4 tumors; both are referred to as “GMB” in the protocol. Participants must be able to understand, read, and speak Danish.

### Recruitment Procedure

Participants will be recruited from the Department of Neuro-oncology at Copenhagen University Hospital in Denmark. The feasibility of the intervention is planned to be evaluated nationally, and the 3 other departments of neuro-oncology in Denmark at Odense University Hospital, Aarhus University Hospital, and Aalborg University Hospital will be invited to participate in the recruitment procedure. A purposive sampling strategy will be applied, and participants will be recruited face-to-face.

### Study Phases

#### Exploratory Phase

A systematic review will identify and explore available evidence of outcomes of supportive group interventions for caregivers to patients diagnosed with a primary brain tumor. To define the group element of an intervention, we decided to include “any kind of intervention, that sought to facilitate some element of interaction between a minimum of two caregivers.” The search will be conducted in the following databases: PubMed, Embase, Web of Science, Emcare, Cochrane Library, and PsycInfo. Peer-reviewed studies in English, German, or Scandinavian will be included without any time limits. The software program Covidence (Veritas Health Innovation Ltd) will support the process of screening [30]. Guidance on the conduct of narrative synthesis in systematic reviews will be used to interpret findings [31]. To address the quality of the included studies, the Mixed Methods Appraisal Tool will be applied [32]. To ensure transparency, the PRISMA (Preferred Reporting Items for Systematic Reviews and Meta-Analyses) guideline will be used [33].

#### Development Phase

According to the MRC framework, the development phase focuses on designing the intervention based on existing evidence and relevant theories, engaging stakeholders, and refining the intervention to ensure contextual fit and relevance to the target population [25]. Therefore, the development of the SUGRI intervention will be grounded in existing evidence from the neuro-oncology field, informed by the findings from the systematic review, and be carried out in close collaboration with stakeholders.

#### PPI Activities

PPI will be central in the development phase, where a national panel of current and bereaved caregivers to patients with a GBM will be established. A panel consisting of 6 to 12 members will allow for deeper interaction and a more trusting environment. Members will be recruited nationally through the Danish Brain Tumor Association. The intervention will be developed and designed in collaboration with a panel of caregivers, using Microsoft Teams as the meeting platform. Online meetings will be scheduled for every second month, and 1 cocreative workshop requiring physical attendance will be planned and facilitated in cooperation with the Danish Knowledge Center for User Involvement in Health Care. The PPI process will be evaluated through a focus group interview with the panel members, guided by the approach described by Krueger and Casey [34]. For the quantitative evaluation, the 22-item Patient Engagement in Research Scale will be used to measure the level of meaningful caregiver engagement in the research process [35,36].

#### Intervention

The intervention will be conducted online and will encompass 2 primary components: an informational element and a group-based element. The final design of the intervention and how the 2 central elements will be incorporated will be developed through an iterative PPI process in cooperation with the panel to integrate existing evidence on caregivers’ needs and preferences. Results from the systematic review from the exploratory phase will also guide the design of the final intervention. The group element will be designed and inspired by existing knowledge of caregiver preferences of supportive group interventions [11,22].

#### Feasibility and Evaluation Phase

The feasibility phase in the MRC framework includes assessment of the feasibility and acceptability of the intervention. It also involves evaluation of the design and the predefined feasibility parameters, addressing uncertainties identified during development, refining the intervention, and considering practical and economic aspects [25]. In the third study phase, the online intervention will be tested on a new group of caregivers, and feasibility will be analyzed and evaluated by integration of qualitative and quantitative methods. A sample size of approximately 30 participants is recommended for feasibility trials [37].

#### Primary Objective and End Points

We hypothesize that offering caregivers a supportive group intervention will empower them in their role by boosting their perceived support, strengthening their family function, and helping them to identify strategies to navigate the challenges of caregiving, ultimately benefiting both caregivers and patients.

The primary objective of this study is to evaluate the feasibility of the SUGRI intervention. The following parameters will be evaluated: recruitment, fidelity, adherence, acceptability, and safety (detailed in Table 1).

**Table 1:** Progression criteria and feasibility parameters.

Study phase	Study	Aim and primary assessment	Data collection or assessment method	Progression criteria or predefined benchmarks
Exploratory phase	Systematic review	Aim: to identify and explore available evidence of outcome of supportive group interventions for caregivers to patients with a primary brain tumor Primary assessment: evidence from peer-reviewed literature	Data synthesis: guidance on the conduct of narrative synthesis in systematic reviews [31] Quality assessment: the Mixed Methods Appraisal Tool [32] PRISMAa guidelines [33]	Proceed to the development phase if: Review confirms a gap for group interventions of the target population Intervention already exists, reconsider scope or adapt intervention Review identifies sufficient evidence to inform group interventions to be positively evaluated
Development phase	PPI^b^ study	Aim: to involve caregivers in the design and development of a supportive group intervention targeted at caregivers to patients diagnosed with a glioblastoma and evaluate the PPI process Primary assessment: caregivers’ experiences and perceived meaningful impact of participation	Qualitative data: focus group interview guidelines by Krueger and Casey [34] Quantitative data: 22-item Patient Engagement in Research Scale [35,36]	Proceed to feasibility and evaluation phase if: A caregiver panel can be successfully established Intervention can be realistically developed in cooperation with the panel, integrating evidence and caregiver input
Feasibility and evaluation phase	Feasibility study	Aim: to test the feasibility of a supportive group intervention among caregivers to patients diagnosed with a glioblastoma Primary assessment: predefined feasibility parameters: Recruitment: is it feasible to recruit participants to the intervention? Fidelity: is the intervention provided as designed? Adherence: how feasible is it to ensure participants adhere to the intervention? Acceptability: what are the experiences and ratings of satisfaction with the intervention? Safety: is the intervention safe for participants?	Qualitative data: semistructured individual interviews with caregivers Quantitative data: patient- and caregiver-reported outcome Quantitative registration of recruitment rate Participants are offered the intervention Quantitative registration of intervention participation and participant feedback via study-specific questionnaires and interviews Participant feedback via study-specific questionnaires and interviews Adverse events from validated questionnaires and interviews	Proceed to development of a stepped care model if: All 5 feasibility parameters meet their predefined benchmarks Estimated recruitment ≥60%c Estimated fidelity 100%c Estimated adherence ≥70%c Estimated acceptability mean ≥4d Safety reported narrativelye

^a^PRISMA: Preferred Reporting Items for Systematic Reviews and Meta-Analyses.

^b^PPI: patient and public involvement.

^c^Recruitment rate, fidelity, and adherence are presented as estimates informed by other caregiver interventions and reported as percentages.

^d^Acceptability is defined as an average score of ≥4 on a 5-point Likert scale in a study-specific questionnaire. The questionnaire includes the item: “Overall, I found the caregiver program to be supportive,” rated from 1=not at all to 5=to a very great extent.

^e^Safety will be reported narratively based on individual postintervention evaluation interviews with caregivers, guided by the following question: “Have you experienced any concerns as a result of your participation that you did not have before?”

The proposed benchmarks for the 5 feasibility parameters have been guided by existing literature on caregiver interventions [38-41]. Predefined progression criteria, as proposed by the MRC framework, will be assessed through a feasibility study aligned with the evaluation design [24]. Moreover, progression criteria are used to guide the decision on whether to proceed to the next phase of the project [25]. Progression criteria for the 3 study phases—the exploratory phase, the development phase, and the feasibility and evaluation phase—are also presented in Table 1.

#### Measurements

Secondary outcomes are patient-reported outcome data completed by caregivers and patients at baseline, postintervention, and follow-up (Table 1). Caregivers will complete the following 3 questionnaires: the Caregiver Roles and Responsibilities Scale to assess broad life impacts for caregivers [42], the Iceland-Family Perceived Support Questionnaire to measure families’ perceived support from nurses and other health care professionals [43], and the Hospital Anxiety and Depression Scale to measure symptoms of anxiety and depression [44]. Patients will complete the following 3 questionnaires: the Functional Assessment of Cancer Therapy-Brain to assess the patients’ health-related quality of life [45]; the MD Anderson Symptom Inventory-Brain Tumor Module to measure patients’ symptom prevalence, intensity, and interference with the daily life [46]; and Hospital Anxiety and Depression Scale [44]. The questionnaires and time assessment are presented in Table 2 and will be collected electronically using Research Electronic Data Capture (REDCap; Vanderbilt University). Demographic data of the participants will be completed at baseline, and information on patient histology will be obtained from the patient’s medical journal and stored in REDCap.

**Table 2:** Measurement tools and assessment time points for the feasibility study.

Outcome measurement tools			Caregiver							Patient			
			T0^a^		T1^b^		T2^c^		T0			T1	T2
**Quantitative**													
	Informed consent	✓^d^						✓					
	Demographic data	✓						✓					
	CRRS^e^ 41 items (0-164 points)	✓		✓		✓							
	ICE-FPSQ^f^ 14 items (14-70 points)	✓		✓		✓							
	HADS^g^ 14 items (0-21 points)	✓		✓		✓		✓			✓		✓
	FACT-Br^h^ 50 items (0-200 points)							✓			✓		✓
	MDASI-BT^i^ 13 core cancer-related symptoms and 9 brain tumor–specific symptoms (0-220 points)							✓			✓		✓
**Qualitative**													
	Semistructured interview			✓									

^a^T0: baseline.

^b^T1: postintervention week 12.

^c^T2: follow-up week 18.

^d^Time points for data collection.

^e^CRRS: Caregiver Roles and Responsibilities Scale.

^f^ICE-FPSQ: Iceland-Family Perceived Support Questionnaire.

^g^HADS: Hospital Anxiety and Depression Scale.

^h^FACT-Br: Functional Assessment of Cancer Therapy-Brain.

^i^MDASI-BT: MD Anderson Symptom Inventory-Brain Tumor Module.

To explore experiences and evaluate the intervention qualitatively, semistructured individual interviews will be carried out with the participating caregivers postintervention. A semistructured interview guide will be applied, and data will be audiotaped and verbatim transcribed.

### Statistical Analysis

Results regarding the feasibility of the intervention and the attainment of predefined feasibility parameters will be presented separately. Feasibility parameters and secondary outcomes, including subscale scores from patient-reported outcomes, will be presented using descriptive statistics. Mean change from baseline to later assessment times will be analyzed using appropriate statistical methods, possibly a linear mixed model to account for repeated measurements on each participant. This will further ensure that no imputation is required to handle missing data under the missing-at-random assumption. No formal adjustment of *P* values to control the number of false positives will be used, as analyses of secondary outcomes should be regarded as exploratory. Patient-reported outcome data will provide valuable insight into the impact of the caring responsibilities, perceived support, and the caregiver role combined with the patient’s well-being, with a particular emphasis on their emotional and functional challenges.

### Qualitative Analysis

Qualitative interview data will be analyzed using thematic analysis, as described by Braun and Clarke [47,48], using the NVivo software (Lumivero) [49]. The interview data will provide the opportunity to evaluate specific elements of the intervention, contributing to a more comprehensive understanding of how different aspects of the intervention impact caregivers. The qualitative and quantitative data will be collected and analyzed separately and then merged. In the overall interpretation, the 2 datasets will be compared, and we will search for potential common patterns [50-52].

### Ethical Considerations

The study adheres to the ethical standards of the institutional and national research committee and complies with the Declaration of Helsinki. The study was approved by the ethical committee of the Capital Region of Denmark (F‑24004933) and registered with the Danish Data Protection Agency (Jr. P‑2022‑739). No compensation is provided for participation. Potential ethical risks include breaches of privacy or integrity, while other types of harm are considered unlikely. Study participants as well as future patients and caregivers may benefit from the study. Measures will be taken to protect integrity. Participants will be enrolled based on oral and written informed consent, which can be withdrawn at any time. All participants will provide written informed consent before enrollment and will be informed about procedures related to confidentiality, safeguarding, and support throughout the intervention. Clear rules for confidentiality will be established at the beginning of the online group sessions, including reminders to not share identifiable information outside the group. To ensure participant well-being, facilitators will be attentive to recognizing signs of emotional distress during the sessions. If a participant appears overwhelmed, they will be offered a one-on-one follow-up conversation and, if necessary, referred to appropriate support services.

### Study Registration

The study is registered on ClinicalTrials.gov (NCT06869577), and the systematic review is registered in PROSPERO (CRD42023392222).

## Results

A national panel of caregivers (N=10) has been successfully established, and the final design of the intervention is currently being developed through an ongoing PPI process. A total of 3 hospitals in Denmark have committed to participating in the recruitment for the feasibility study. Recruitment began in April 2025. The multicenter feasibility assessment of the SUGRI intervention is scheduled to start in autumn 2025, and the final evaluation is planned for spring 2026

## Discussion

### Positioning Supportive Caregiver Care Within the Neuro‑Oncology Landscape

Caregiver support within the neuro-oncology field remains suboptimal. This paper describes a study protocol for a supportive group intervention for caregivers to patients diagnosed with a GBM. The intervention is designed to address the challenges faced by caregivers, with particular emphasis on their need for information and peer support. We expect that participation in a group intervention will empower caregivers by enhancing their perceived social support, strengthening family functioning, and helping them identify strategies to navigate caregiving challenges—ultimately benefiting both caregivers and patients.

Caregivers may benefit from continuous access to support throughout the patient’s treatment trajectory, tailored to address the specific emotional challenges and stressors that caregivers face in their role as a caregiver [53]. Previous interventions have been carried out using a dyadic approach, where patients and caregivers receive the intervention together, allowing for mutual support [54,55]. However, Mulbury et al [56] examined preferences for intervention delivery, comparing a dyadic format, involving both patient and caregiver, with a caregiver-only format and found that the caregiver-only approach was significantly more beneficial for caregivers. This supports the findings of Mallya et al [22], who reported a clear preference among caregivers for dedicated support groups separate from those involving the patient. Group-based interventions offer caregivers a chance to connect with others in similar situations, creating a safe and supportive environment for sharing emotions, concerns, and experiences [13,22]. Peer support may also provide valuable practical advice, help normalize experiences, and offer new perspectives on personal caregiving experiences [13,14]. Group-based interventions may also offer validation, a sense of belonging, and foster hope [4].

Given the diverse nature of caregiver needs, shaped by demographic, course of the disease, and patient symptom burden, future research may benefit from moving beyond standardized approaches and explore the potential of multicomponent interventions [14,22]. Therefore, the MRC framework may provide an overall structured framework designed to guide this study. An intervention may be described as complex when it includes multiple components interacting with each other and allows for flexibility or tailoring [25]. The SUGRI intervention is planned to include 2 primary components: an informational element and a group-based element. Integration of these 2 components will be developed through a PPI process.

The updated MRC framework from 2021 includes 6 core elements to be addressed throughout the research process, one of which is engaging stakeholders [24]. Engagement of the stakeholders meaningfully at every stage of the research process is essential to develop interventions that positively impact health outcomes for patients and caregivers [24]. In the SUGRI project, we plan to establish a PPI collaboration with a panel of current and bereaved caregivers throughout the project. The MRC framework recommends involving stakeholders early in the research process [25]; however, out of respect for the caregivers’ time, we have chosen not to include them at the very beginning during the protocol stage. The protocol, while serving as the foundation for the project, is intentionally described in broad terms, as the detailed design of the intervention will be developed collaboratively with the caregiver panel. Given the poor prognosis of a GBM, we anticipate the panel to be dynamic, with caregivers leaving as patients enter end-of-life phases and new caregivers are invited to join. Most of the panel activities are scheduled to be online, as caregivers may have practical difficulties to schedule physical meetings due to their caregiver role.

The feasibility test of the SUGRI intervention aims to address caregivers’ supportive needs throughout the patient’s disease and treatment trajectory. Moreover, we anticipate the SUGRI intervention to inform how a tailored intervention can be both feasible and meaningful within the context of caregivers’ daily lives.

### Strengths and Limitations

A major strength of the project is that it addresses an evident and urgent need for support among caregivers of patients with GBM, a need that has also been expressed by clinicians seeking structured ways to assist caregivers in navigating this profoundly life-changing situation. A limitation of this protocol is that the intervention has not yet been fully developed. Consequently, there is uncertainty regarding its specific content, structure, and timing, which will determine the feasibility and overall evaluation of the intervention.

The study incorporates PPI, which represents a methodological strength by ensuring the intervention is tailored to the target population and enhances its relevance and acceptability. The project also includes an evaluation of the PPI process itself, thereby contributing to the validation of the intervention from the perspective of the involved caregivers.

The guidance of the MRC framework is another strength, as it provides a structured and evidence-informed approach to intervention development. It supports transparency, theoretical grounding, and relevance to the target population, thereby enhancing the quality and potential impact of the final intervention.

### Future Directions

Health care resources should be used in an efficient manner to ensure the greatest impact. At the same time, it is essential that support efforts are tailored to the individual needs, particularly in light of the evolving shortages in the health workforce [17]. Moreover, caregiver support is likely to be cost-effective, providing societal benefits by improving health outcomes. Cost-benefit of caregiver intervention is not part of this protocol but would be interesting to evaluate.

Stepped care is a health care delivery model that allocates resources efficiently by matching the level of care to the individual’s needs [57,58]. Depending on the design of the SUGRI intervention, a stepped care model may be beneficial for the future direction of the study. In a stepped care approach, the intensity of support provided to caregivers can be adjusted according to their individual needs and preferences, ensuring that they receive appropriate and timely support. Applying a stepped care model to future caregiver interventions may enhance their adaptability to hospital workflows and better address the individual needs of caregivers.

### Dissemination Plan

Dissemination of results includes publication of articles in peer-reviewed journals: a systematic review, a PPI article, and an article on the feasibility of the intervention. Moreover, results will be presented at scientific conferences, to clinicians within the neuro-oncology field, and to the panel of caregivers who participated in the PPI process.

### Conclusions

The SUGRI study will develop, test, and evaluate the feasibility of a supportive group intervention. The intervention has the potential to address specific caregiver needs and strengthen their supportive network by offering a supportive group intervention to the caregivers. Only few group interventions targeting the neuro-oncology caregiver have been carried out, and results may contribute with new internationally relevant knowledge to improve conditions and care for patients with GBM and their caregivers. Providing caregivers with support may enhance their perceived support, strengthen family functioning, and provide them with strategies to manage caregiving challenges, ultimately benefiting both caregivers and patients.

### Clinical Implications

Family members of patients diagnosed with a GBM often assume caregiver roles and take on a great responsibility. The SUGRI study accepts this fact and assumes caregivers not only not only need support but also represent an untapped resource. An online intervention may enhance support for caregivers, offering flexibility and helping to reduce the demand on clinical staff. This approach underscores the need for labor-saving technologies to address the nursing shortage and improve patient care [17]. To the best of our knowledge, a group intervention has not yet been systematically offered to caregivers of patients with a GBM in a Danish context, making its feasibility and outcomes of significant interest to the field of neuro-oncological caregiver research.

## Appendix

Multimedia Appendix 1 SPIRIT 2025 checklist of items to address in a randomized trial protocol.

## Abbreviations

GBM: glioblastoma
MRC: Medical Research Council
PPI: patient and public involvement
PRISMA: Preferred Reporting Items for Systematic Reviews and Meta-Analyses
REDCap: Research Electronic Data Capture
SPIRIT: Standard Protocol Items: Recommendations for Interventional Trials
SUGRI: Supportive Group Intervention for caregivers to patients diagnosed with a glioblastoma
